# Weighted correlation network analysis identifies multiple susceptibility loci for low‐grade glioma

**DOI:** 10.1002/cam4.5368

**Published:** 2022-10-28

**Authors:** Xiaodong Niu, Qi Pan, Qianwen Zhang, Xiang Wang, Yanhui Liu, Yu Li, Yuekang Zhang, Yuan Yang, Qing Mao

**Affiliations:** ^1^ Department of Neurosurgery and West China Glioma Center West China Hospital, Sichuan University Chengdu China; ^2^ Department of Dermatology Chongqing Hospital of Traditional Chinese Medicine Chongqing China; ^3^ Department of out‐patient West China Hospital, Sichuan University Chengdu China; ^4^ Department of Anesthesia West China Hospital, Sichuan University Chengdu China

**Keywords:** DNA methylation, Low‐grade glioma (LGG), mRNA expression, WGCNA

## Abstract

**Background:**

The current molecular classifications cannot completely explain the polarized malignant biological behavior of low‐grade gliomas (LGGs), especially for tumor recurrence. Therefore, we tried to identify suspicious hub genes related to tumor recurrence in LGGs.

**Methods:**

In this study, we constructed a gene‐miRNA‐lncRNA co‐expression network for LGGs by a weighted gene co‐expression network analysis (WGCNA). GDCRNATools and the WGCNA R package were mainly used in data analysis.

**Results:**

Sequencing data from 502 LGG patients were analyzed in this study. Compared with recurrent glioma tissues, we identified 774 differentially expressed (DE) mRNAs, 49 DE miRNAs, and 129 DE lncRNAs in primary LGGs and ultimately determined that the expression of MKLN1 was related to tumor recurrence in LGG.

**Conclusion:**

This study identified the potential biomarkers for the pathogenesis and recurrence of LGGs and proposed that MKLN1 could be a potential therapeutic target.

## Graphical abstract

In this study, we constructed a gene‐miRNA‐lncRNA co‐expression network for low‐grade gliomas (LGGs) by a weighted gene coexpressionnetwork analysis (WGCNA). This study identified the potential biomarkers for the pathogenesis and recurrence of LGGsand proposed that MKLN1 could be a potential therapeutic target.

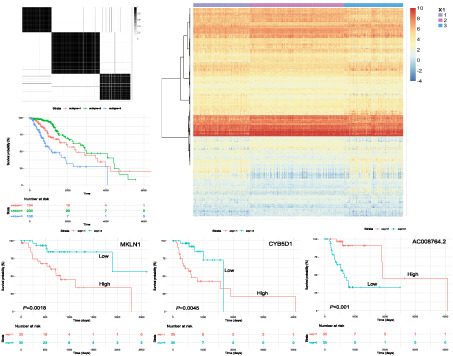

## 1 INTRODUCTION

Typical low‐grade gliomas (LGGs), World Health Organization (WHO) grade 2 gliomas, are generally slowly growing and locally infiltrative brain tumors, and usually occur in young or middle‐aged adults.^1^, ^2^ Most patients who received comprehensive treatments (including maximal surgical resection and follow‐up chemoradiotherapy based on molecular neuropathology) may have better survival, but these types of tumors are unlikely to change their invasive nature.^3^, ^4^ Recent studies disclosed that LGGs were more concordant with molecular status (IDH, 1p/19q, and TP53) than with histologic class.^4^, ^5^, ^6^ LGGs with IDH wild‐type, 1p/19q non‐codeletion, and TERT mutation can progress to more aggressive gliomas, such as anaplastic glioma and secondary glioblastoma (GBM).^7^ However, these molecular classifications cannot completely explain all the malignant biological behaviors of low‐grade gliomas. Especially for tumor recurrence. Therefore, using new theory and higher‐dimensional data is significant to supplement the regulatory mechanism of low‐grade gliomas.

The competing endogenous RNA (ceRNA) hypothesis has the potential to account for the functions of yet uncharacterized RNAs, including lncRNAs, miRNAs, and mRNAs.^8^, ^9^, ^10^, ^11^, ^12^, ^13^, ^14^ Recent efforts have identified a series of novel genes by identifying complex lncRNA regulatory networks in glioblastoma, including lncRNA MCM3AP‐AS. In addition, lnRNAs‐SOX21‐AS1, RP6‐99 M1.2, RP11‐375B1.3, CTD‐2127H9.1, and RP3‐449 M8.9 predicted survival independent of all other markers.^15^ Studies, including our previous work,^16^ have yielded growing evidence of the importance of ceRNA networks in GBMs.^17^, ^18^ However, comprehensive analyses for combining lncRNA, miRNA, and mRNA expression data of low‐grade gliomas are still scarce. Furthermore, the association of the identified biomarkers with clinical characteristics, such as driver genes for the recurrence of LGGs, remains largely unknown.

In this study, we constructed a gene‐miRNA‐lncRNA co‐expression network for LGGs by a weighted gene co‐expression network analysis (WGCNA) and identified the modules related to external information to finally identify the key driver genes in interesting modules related to the recurrence of LGGs. We aimed to complement the characteristics of the lncRNA‐miRNA‐mRNA network in LGGs and ultimately identify multiple susceptibility loci and hub genes for LGGs.

## 2 METHODS

### 2.1 Patient information acquisition and data preprocessing

The *GDCRNATools* R package was used to obtain patient data from the National Institutes of Health (NIH)^19^ and Genomic Data Commons database (GDC, https://gdc.cancer.gov/) and to process these data. Briefly, *GDCRNATools* were used to process the Isoform Expression Quantification (BCGSC miRNA Profiling) data, Gene Expression Quantification (HTSeq‐Counts) data, and Clinical (Clinical Supplement) data for the downstream analysis, such as differential gene expression analysis (DE analysis), Hypergeometric test, and Pearson correlation analysis. Finally, this *R* package can allow researchers to perform analyses by simply running a few functions and integrating their pipelines, such as molecular subtype classification and WGCNA.

### 2.2 Weighted gene correlation network analysis

The WGCNA R software package was used for finding clusters/modules of highly correlated genes.^20^ With the help of WGCNA, we aimed to establish a network of differentially expressed lncRNA/miRNA/mRNA according to the ceRNA hypothesis. Briefly, according to the WGCNA protocols, (1) 502 low‐grade glioma patients' data were input and cleaned; (2) the gene network and identification of modules were constructed step‐by‐step via Topological Overlap Matrix (TOM); (3) modules were related to external clinical traits, and important genes were identified; (4) network analysis was interfaced with other data such as functional annotation and gene ontology. Generally, the threshold of height was 0.95, while that of the minimal module size was 20 genes for the resultant dendrogram.

### 2.3 Survival analysis and prognostic test evaluation for biomarkers

The molecular, clinical, survival, and histopathological data were acquired from the Genomic Data Commons database (GDC) (https://gdc.cancer.gov/). A total of 502 LGG patients were enrolled in this study. All analyses were determined to be statistically significant, with *p* < 0.05. The plotted survival curves were constructed by *survival and survminer* R packages. In our study, we tried to use the receiver operating characteristic (ROC) curve to evaluate the best genes. The *plotROC* R package was used to generate the ROC curve.

### 2.4 Co‐expression correlation

The criteria for correlation analysis are as follows: the expression values of genes from RNA‐seq data were scaled with log2 (FPKM +0.01), and the expression data of glioma were downloaded from the TCGA project via Genomic Data Commons Data Portal. The co‐expression correlation results were obtained from *starBase v3.0* (http://starbase.sysu.edu.cn/panCancer.php). *starBase v3.0* is an open‐source platform for studying the miRNA‐mRNA, miRNA‐ncRNA, ncRNA–RNA, RBP‐mRNA, and RBP‐ncRNA interactions from the degradome‐seq, CLIP‐seq, and RNA–RNA interactome data. Gene co‐expression was found under *the starBase Pan‐Cancer Analysis Platform*.

### 2.5 Gene ontology (GO) and Kyoto encyclopedia of genes and genomes (KEGG) pathway analysis

To determine the biological mechanisms of the cohort of enrolled glioma patients, the differentially expressed genes (DEG) were subjected to GO and KEGG pathway analysis. KEGG enrichment analysis was imported into the KEGG database. The *clusterProfiler* R package was used to classify GO functions and to generate the GO and KEGG plots.

## 3 RESULTS

### 3.1 Patient information acquisition and data preprocessing

**FIGURE 1:**
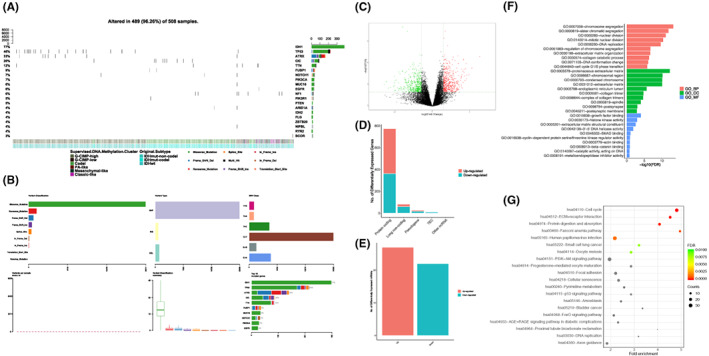
Mutational landscape of low‐grade gliomas (LGGs). (A) Oncoplot of the top 20 most frequently mutated genes in the LGGs. (B) Mafplot of the included patients, which displays the number of variants in each sample as a stacked barplot and variant types as a boxplot summarized by Variant_Classification. (C) Volcano plot highlighting significant genes. (D, E) Plot for differentially expressed coding and non‐coding RNAs between primary and recurrence LGGs. (F, G) The KEGG and GO analysis for the DE mRNAs.

A total of 502 LGG patients were included in this study. To visualize multiple genomic alteration events of the overall situation of the included patients, we drew an Oncoplot (Figure 1A) and a MAFplot (Figure 1B) using the Bioconductor package of maftools (Mayakonda and Koeffler 2016).^21^ Similar to previous studies, the most frequent mutation in LGG was IDH (77%), and the most common mutation type was C > T. As disclosed in Figure 1C, A total of 952 mRNAs, miRNAs, and lncRNAs exhibited differential expression (DE) between primary and recurrent low‐grade gliomas. Subsequently, compared with recurrent glioma tissues, we further identified 774 DE mRNAs, 49 miRNAs, and 129 lncRNAs, including 18 upregulated and 51 downregulated lncRNAs, 82 downregulated lncRNAs and 47 upregulated lncRNAs (log2 [fold change] > 0.58, *p* < 0.05) (Figure 1D), including 22 downregulated miRNAs and 27 upregulated miRNAs (log2 [fold change] > 0.58, *p* < 0.05) (Figure 1E). The results of the KEGG and GO analyses for the DE mRNAs are displayed in Figure 1F,G.

### 3.2 Survival subtypes based on lncRNA, miRNA, and mRNA expression profiles

**FIGURE 2:**
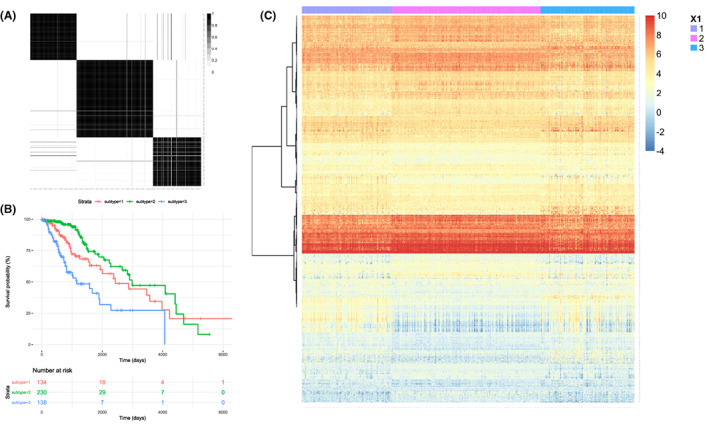
(A) Non‐negative matrix factorization analysis for included patients. (B) Survival curves of the enrolled patients, the grouping information was based on the NMF analysis (*p* = 0.02). (C) Heatmap of the difference mRNA expression for the groups.

To identify the potential DE lncRNAs, DE miRNAs, and DE mRNAs that could predict the overall survival (OS) period of LGGs, we used *IntNMF* R package to conduct nonnegative matrix factorization (NMF) cluster analysis, and the final cluster number *k* was set as *K* = 3 (Figure 2A). In addition, the overall survival analysis of the three clusters was drawn with a Kaplan–Meier plot (Figure 2B), and the heat map of these clusters showed distinct differences among groups (Figure 2C). As demonstrated in Figure 2B, subtype 3 (*X* = 3, *n* = 138) showed an obvious dismal prognosis compared with subtypes 1 (*n* = 134) and 2 (*n* = 230).

### 3.3 WGCNA for 3 subtypes in LGG patients

**FIGURE 3:**
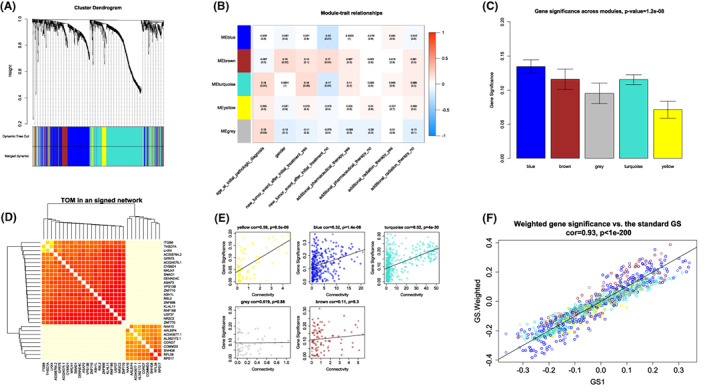
WGCNA analysis for 3 subtypes in LGG patients. (A) Hierarchical cluster analysis was used to detect co‐expression clusters and each color represented a module. (B) Heat map contained the *p*‐value correlation from the linear mixed‐effects model. The MEblue module was highly negatively related to new events after tumor initial treatment. (C) Bar plot of mean gene significance. (D) Heatmap showed the Topological Overlap Matrix (TOM) of genes. Red color represents higher overlap and light blue color represents lower overlap. (E) Correlation between each module membership and gene significance. (F) Network‐weighted gene significance (*y*‐axis) was shown against the standard (input) gene significance (*x*‐axis). The weighted gene significance measure was amplified for genes with high module significance.

To identify the key lncRNA, miRNA, and mRNA drivers that induce fast recurrence in LGG, we used the *WGCNA* R package to analyze the DE lncRNAs, DE miRNAs, and DE mRNAs among primary LGG and recurrent LGG (DE lncRNAs, DE miRNAs, and DE mRNAs = 952, Figure 1C). A total of 5 models were constructed by clustering the dissimilarity based on the consensus topological overlap (Figure 3A). As a result, based on the relationships of modules with the clinical characteristics of LGG glioma, such as gender, new tumor events, and additional pharmaceutical therapy, we confirmed from the results that the MEblue module was highly negatively related to new tumor events after initial treatment (*r* = −0.22, *p* = 0.01, Figure 3B). A barplot of module significance was used to define mean gene significance in the module. The blue module was the most promising (Figure 3C). We next drew a heatmap among the 30 most significant genes identified from the blue module (Figure 3D). We also calculated intramodular connectivity (x‐axis) vs. gene significance (y‐axis) plotted separately for each module (Figure 3E). The results were shown in Figure 3F, where network‐weighted gene significance (y‐axis) was shown against the standard (input) gene significance (x‐axis). The weighted gene significance measure was amplified for genes with high module significance.

### 3.4 Gene co‐expression network for WGCNA

**FIGURE 4:**
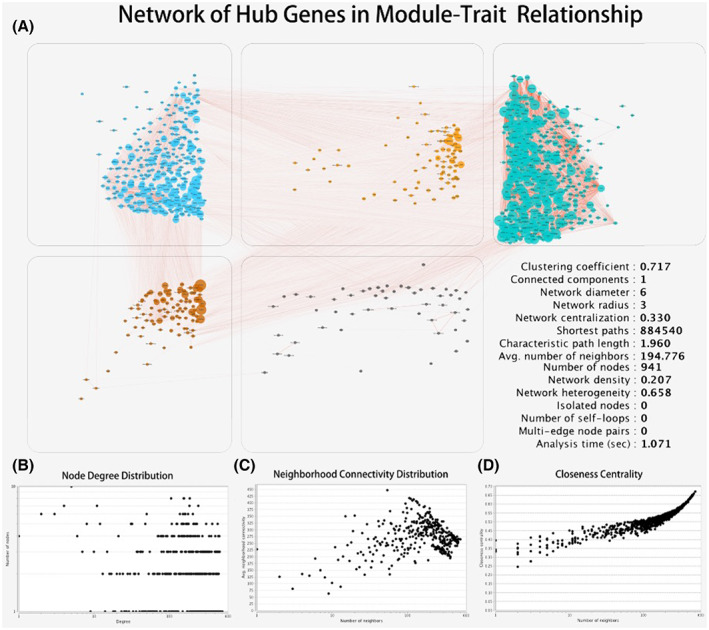
(A) An overview of the relationship between DE genes among different modules is present, and (B–D) the interconnectivity was high among the modules.

**FIGURE 5:**
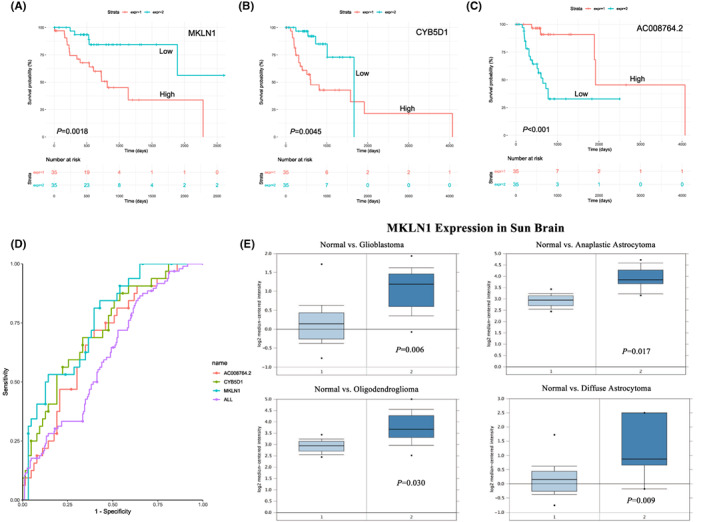
Clinical analysis of hub‐genes from WGCNA results. (A) Overall survival (OS) analysis for patients with MKLN1 difference expression (*p* = 0.0018). (B) OS with CYB5D1 difference expression (*p* = 0.0045). (C) OS with AC008764.2 difference expression (*p* < 0.001). Red lines represent the high expression of mRNA, and light blue lines represent the low expression of mRNA. (D) Prognostic accuracy of the signature evaluated by the AUCs. (E) Validation of MKLN1 expression level in the Sun Brain dataset patient population.

An overview of the correlation between DE genes among different modules is presented in Figure 4A, and the interconnectivity was high among the modules (Figure 4B–D). We further analyzed the visualized gene network in MEblue, which is related to new tumor events (Figure 5). The hub genes in the modules showed a strong connection, which suggested the potential coregulation of the involved processes. After the final screening, we focused on the following hub genes: SMAD1, MKLN1, CYB5D1, AC024075.1, GPR75, AC008764.2, LHX4, THSD7A, ITGB8, and DENND4C.

### 3.5 Clinical implications for biomarkers from WGCNA biomarkers

Subsequently, we analyzed the abovementioned 10 hub genes in the GDC subtype Clust3 cohort (*n* = 138). First, for each of the hub genes, the data were categorized into high and low groups using expression quartiles (*n* = 36/37 for each group). By using the ROC curve method, we identified the top three hub genes (MKLN1, CYB5D1, AC008764.2) with optimal sensitivity and specificity for discrimination between dead and alive patients. Kaplan–Meier log‐rank tests demonstrated that the high expression of MKLN1 and CYB5D1 indicated poor prognosis in the subtype Clust3 cohort but showed better survival for AC008764.2 than the counterparts **(**Figure 5A–C **)**.

We also checked whether the expression level of MKLN1, which holds the most optimal sensitivity and specificity in the AUC curve (Figure 5D), may correlate with the tumor pathologic stage. By utilizing the Sun Brain cohort from the database cbioportal (http://www.cbioportal.org), we found that MKLN1 expression levels in all tumor groups were higher than those in normal counterparts (Figure 5E).

## 4 DISCUSSION

Typical LGGs are defined as WHO grade II primary brain neoplasms; unlike the poor overall survival of glioblastoma, the median survival of LGGs ranges from 1 year to 15 years.^1^, ^2^ In recent years, many biomarkers, such as IDH1/IDH2, MGMT, TP53, TERT, and 1p/19q, have prognostic significance^22^, ^23^; however, the existing molecules could not completely explain all the malignant biological behaviors of low‐grade gliomas.^7^, ^24^ For this reason, we conducted WGCNA for low‐grade patients to make a molecular diagnosis supplement for LGG patients. The enrolled data were from 519 low‐grade glioma patients. Incorporating 97 primary solid tumor samples and metastatic LGG samples using the GDC LGG dataset, we further analyzed 774 DE mRNAs, 49 DE miRNAs, and 129 DE lncRNAs. Moreover, a potentially novel prognostic biomarker, MKLN1, was identified from the ceRNA network built by the WGCNA method.

One of the major goals of our study was to use bioinformatic methods to explain the molecular reason that induces polarized prognosis in LGG patients. The subtypes were classified into three groups (*k* = 3, Figure 2) because the survival curve for each group could be separated clearly when *K* = 3. We chose the worst prognosis group for further analysis and tried to determine which hub‐gene could affect the prognosis most.

WGCNA is a powerful tool for performing various aspects of weighted correlation network analysis, which was developed by Langfelder and Horvath.^20^ To date, this method has been applied to many kinds of diseases.^25^, ^26^, ^27^, ^28^, ^29^ For instance, Zhai X and his colleagues used WGCNA co‐expression network analysis to identify colon cancer recurrence‐associated genes,^28^ and Maertens A used WGCNA to reveal novel transcription factors (TF) associated with a dose–response of bisphenol.^29^ To our knowledge, our research first used WGCNA to identify risk recurrence genes in LGG patients. In our research, the DE lncRNAs, DE miRNAs, and DE mRNAs associated with tumor recurrence in MEbrown were further analyzed.

Further survival and ROC curve analysis of hub genes from WGCNA results revealed that the top three hub genes (MKLN1, CYB5D1, AC008764.2) were identified. MKLN1 and CYB5D1 had an oncogene role and the high expression indicated poor prognosis in the subtype Clust3 cohort, however, AC008764.2 was associated with a good prognosis. Among them, MKLN1 holds the most optimal sensitivity and specificity in the AUC curve. MKLN1 (Muskelin 1) is an intracellular protein mediating cell spread and cytoskeletal responses to the extracellular matrix component thrombospondin I (MIM 188060).^30^, ^31^, ^32^ Recent studies have reported that MKLN1 is involved in many diseases,^31^, ^32^, ^33^ and a meta‐analysis genome‐wide association study suggested that MKLN1 is related to early‐onset bipolar disorder,^33^ and Tagnaouti et al.^33^ reported that muskelin are expressed throughout the central nervous system (CNS) with significantly high levels in the hippocampus and cerebellum regions, suggesting that MKLN1 is a multifunctional protein associated with cell membranes and/or large protein complexes in most neurons. Moreover, MKLN1‐MET fusion was found in IDH‐mutant GBMs with G‐CIMP‐demethylated profile and can promote the proliferation and progression of glioma.^31^, ^34^ The MKLN1 expression level of the different glioma types in the Sun Brain dataset was consistent with previous studies. However, there are no relative studies on MKLN1 in LGGs. In the present study, we used bioinformatics analysis to find that high MKLN1 expression in LGGs is associated with a poor prognosis and associated with tumor recurrence in LGGs, which has enriched the knowledge of MKLN1 dysfunction. At present, the biological mechanisms of MKLN1 in LGG are being studied. In addition, another hub gene CYB5D1 (cytochrome b5 domain containing 1) is a heme‐binding protein, which can enable metal ion binding. A recent study identified that CYB5D1 may function in a redox signaling pathway to coordinate ciliary beating. However, the functions and mechanisms of CYB5D1 in cancers, including glioma, are still unknown. In this study, the results showed that high CYB5D1 expression in LGGs is associated with a poor prognosis. At present, the biological mechanism of MKLN1 and CYB5D1in LGG are being studied.^35^

## 5 CONCLUSION

In the present study, we constructed a ceRNA network and identified novel biomarkers, especially the MKLN1 gene, that have the potential for prognosis prediction in LGG patients. We hope that our research could provide a stratification marker for survival outcomes and could be a potential therapeutic target for LGGs. The biological mechanism by which dysfunction of the MKLN1 gene affects LGGs is expected in future research.

## AUTHOR CONTRIBUTIONS

**Xiaodong Niu:** Conceptualization (lead); data curation (lead); funding acquisition (equal); resources (equal); writing – original draft (equal); writing – review and editing (equal). **Qi Pan:** Conceptualization (equal); funding acquisition (equal); resources (equal); software (equal). **Qianwen Zhang:** Data curation (equal); resources (equal). **Xiang Wang:** Supervision (equal); writing – review and editing (equal). **Yan‐Hui Liu:** Conceptualization (equal); supervision (equal); writing – review and editing (equal). **Yu Li:** Conceptualization (equal); supervision (equal); writing – review and editing (equal). **Yuekang Zhang:** Conceptualization (equal); supervision (equal); writing – review and editing (equal). **Yuan Yang:** Conceptualization (equal); funding acquisition (equal); software (equal); supervision (equal); writing – original draft (equal); writing – review and editing (equal). **Qing Mao:** Conceptualization (equal); funding acquisition (equal); resources (equal); supervision (equal); writing – review and editing (equal).

## FUNDING INFORMATION

This work was supported by the National Natural Science Foundation of China (81902532, 81904218, 82072773, and 82203456), the fellowship of China Postdoctoral Science Foundation (2022M712255), and the Spark Project of Sichuan University (No. 2082604401223).

## CONFLICT OF INTEREST

The authors have no conflict of interest to declare.

## CONSENT FOR PUBLICATION

All authors have read and approved the manuscript.

## ETHICS APPROVAL AND CONSENT TO PARTICIPATE

Not applicable.

## Data Availability

Research data are obtained freely from the National Institutes of Health (NIH) and Genomic Data Commons database (GDC, https://gdc.cancer.gov/).
